# Non-Linear Population Firing Rates and Voltage Sensitive Dye Signals in Visual Areas 17 and 18 to Short Duration Stimuli

**DOI:** 10.1371/journal.pone.0002673

**Published:** 2008-07-16

**Authors:** David Eriksson, Tamas Tompa, Per E. Roland

**Affiliations:** Brain Research, Department of Neuroscience, Karolinska Institute, Solna, Sweden; Baylor College of Medicine, United States of America

## Abstract

Visual stimuli of short duration seem to persist longer after the stimulus offset than stimuli of longer duration. This visual persistence must have a physiological explanation. In ferrets exposed to stimuli of different durations we measured the relative changes in the membrane potentials with a voltage sensitive dye and the action potentials of populations of neurons in the upper layers of areas 17 and 18. For durations less than 100 ms, the timing and amplitude of the firing and membrane potentials showed several non-linear effects. The ON response became truncated, the OFF response progressively reduced, and the timing of the OFF responses progressively delayed the shorter the stimulus duration. The offset of the stimulus elicited a sudden and strong negativity in the time derivative of the dye signal. All these non-linearities could be explained by the stimulus offset inducing a sudden inhibition in layers II–III as indicated by the strongly negative time derivative of the dye signal. Despite the non-linear behavior of the layer II–III neurons the sum of the action potentials, integrated from the peak of the ON response to the peak of the OFF response, was almost linearly related to the stimulus duration.

## Introduction

In the mammalian eye there are ON center and OFF center ganglion cells providing two major pathways to the lateral geniculate nucleus [1]. From the lateral geniculate nucleus the ON and OFF pathways connect to the primary visual area, area 17. The sudden increase in luminance by a stimulus against the visual background is encoded by the retina as an increase in the firing of the ON center ganglion cells and the offset of such a stimulus is encoded as an increase of the OFF center ganglion cells [1]. The increase in luminance contrast and the decrease in luminance contrast then is faithfully and rapidly signaled by ON and OFF increases in the firing rate of the neurons in area 17 [2]–[4]. Thus the duration of a stimulus should be encoded already at the retinal ganglion cells, as the time interval between the ON firing and the OFF firing will tell the duration of any transient stimulus. However, humans have difficulties discriminating the durations of stimuli lasting less than 100 ms [5], [6]. Furthermore, short stimuli lasting less than 100 ms are perceived as lasting longer than they actually do. This phenomenon is called visual persistence and has puzzled scientists for more than 200 years [7]. A closer look of the responses of the retinal ganglion cells (cat), shows that when the duration of a stimulus is less than 70 ms, the ON retinal ganglion cells fire for 60–70 ms no matter how short the stimulus is [8]. This effect is not due to changes in the intensity of the stimuli [8], [9].

In the lateral geniculate nucleus, the ON neurons fire on the average with somewhat shorter durations to short luminance contrast stimuli (50 ms to stimuli of 40 ms duration; [10]). The OFF neurons though fire with longer latencies some 50–60 ms after the stimulus offset and with longer durations (often more than 100 ms; [10], [11]). This pattern is repeated by the neurons in layer IV of area 17, receiving the axons from the lateral geniculate nucleus [11]–[13]. Simple and complex cells in layer IV fire with short latency ON responses and delayed OFF responses to short duration luminance contrast stimuli [10], [14].

These single unit studies indicated non-linearities in the timing of ON and OFF responses in the visual cortex, and that these non-linearities to some extent may be the effect from local cortical inhibition [14]–[16]. As the layer IV neurons mostly send synapses to layers III and II [17], [18], the layers II–III may be important for fast computation of incoming visual signals. The computation in layers III and II of the messages from layer IV may be of some importance, especially as layer III neurons in area 17 send axons to other, higher order, visual areas [19]–[21]. The layer II and III neurons though seem to respond briefly to static stimuli of short duration, but the responses are variable and the number of single neurons studied is small [14]. In general, single neurons in area 17 have a large variation in their ON and OFF latencies and in the number of action potentials evoked by stationary luminance contrast stimuli [10], [22]–[24]. Although single units may show important properties in their membrane potentials and variable firing, one must examine the firing and membrane potential changes of large populations of neurons to reveal the importance of these properties for the brain.

We consequently recorded the changes in the firing rate and membrane potentials of populations of neurons in layers II and III when the eye was exposed to stationary luminance contrast stimuli of different duration. Here we examine the *null* hypothesis that the OFF responses to stimuli of short duration do not depend on the instantaneous membrane potentials and firing rates of the layer II–III neurons at stimulus offset. Our hypothesis thus is that the membrane potential and firing rate exactly at stimulus offset will determine the time interval between the ON and OFF response. We used a small square of different durations with a luminance higher than the surrounding display as stimuli. To exclude the influence of luminance contrast of the ON and OFF responses all stimuli had identical contrast.

**Figure 1:**
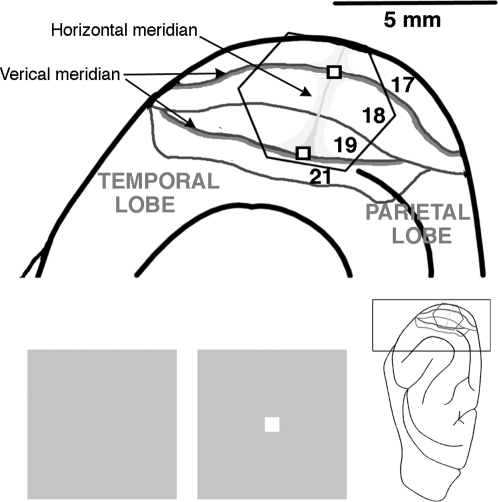
Experimental setting. Insert shows the right hemisphere of the ferret brain. The visual cortex is enlarged at the left. The 464 channel hexagonal photodiode array monitors visual areas 17, 18, 19 and 21. In all animals, the left visual cortex was examined, but because the photodiode array flips the image of the cortex the enlarged picture of the visual cortex here corresponds to what is shown in the other Figures. Each channel of the array picks up the signal from a cortex spot with a diameter of approximately 150 µm. The representations of the vertical meridians and the horizontal meridian of the field of view are shown. The estimated retinotopic position of the 2.5° square used as stimulus are also shown. Bottom: Experimental conditions. Left: the stimulus condition: the luminance contrast square on the gray background; right: the control condition; the gray background.

We recorded the action potentials of multiunits in layers I–III of the ferret visual areas 17 and 18 and combined this with measurements of a voltage sensitive dye (VSD) signal [25]–[27] (**Fig. 1**).

### Explanatory model

In *vitro*, the optical signal of the VSD has excellent linearity with the membrane potential, Vm, within a range of ±300 mV [28]. The dye responds extremely fast, less than 1 µs to any change in the membrane potential [28]. In *vivo*, the VSD binds to the membranes of the cells in cortex and reliably follows their membrane potentials. However, the stained membranes of dendrites, un-myelinated axons, and cell bodies are mixed in a mesh. Furthermore, the photons from the deeper layers of cortex are attenuated and those from the superficial layers are scattered to some extent. This implies that in vivo one can only detect the VSD signal from layers I–III of the cortex [29]–[33]. For each small cortical volume, we measure the difference signal of the voltage sensitive dye between a gray screen with a small luminance contrast square and the control, a gray screen. As the VSD signal depends on the amount of dye used, we calculate the VSD signal, henceforth called ΔV(t), as the difference in fluorescence to the stimulus minus the fluorescence to the baseline gray screen, divided by the fluorescence obtained when the screen is black. The ΔV(t) is not measured in mV, therefore the ΔV(t) only measures the *relative* changes in membrane potentials of the layer I–III neurons in vivo [29]–[34]. In vivo the neurons are slightly depolarized from −65 mV to −60 mV, even under the anesthesia that is used in our experiments [29]–[31], [35]. Surprisingly, when an animal is stimulated with a sensory stimulus, the ΔV(t) closely follows the time course of the Vm averaged over a population of neurons [29]–[31]. This means that the relation between the Vm and the ΔV(t) is approximately linear during physiological stimulus conditions.

In the explanatory model, for each small cortical volume, we regard all stained cells with membranes in layers I–III as *one compartment*. In this compartment all cell membranes create a capacitance, Cm, keeping the ionic imbalance between the extracellular fluid and the intracellular fluid. As all neurons have the same specific capacitance, we can regard the capacitance of the compartment as one, constant value Cm. According to the equation for a capacitor
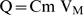
(1)In which V_M_ is the average membrane potential of a population of neurons and Q is the charge.

Taking the derivative and rearranging
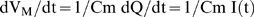
(2)in which I(t) is the net inward current.

As the ΔV(t), the relative change in population membrane potential, experimentally is linearly related to the V_M_ [29]–[31], we can substitute V_M_ by ΔV(t)
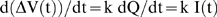
(3)in which k is a constant.

From equation (3) it follows that d(ΔV(t))/dt is proportional to the net inward current in the layer I–III compartment.

For stimulus durations shorter than 133 ms, we found non-linear changes in the timing, duration and amplitudes of both ON and OFF responses in both population firing and population membrane potentials. These non-linearities could be explained by a strong decrease in the net inward current at the stimulus offset.

## Results

**Figure 2:**
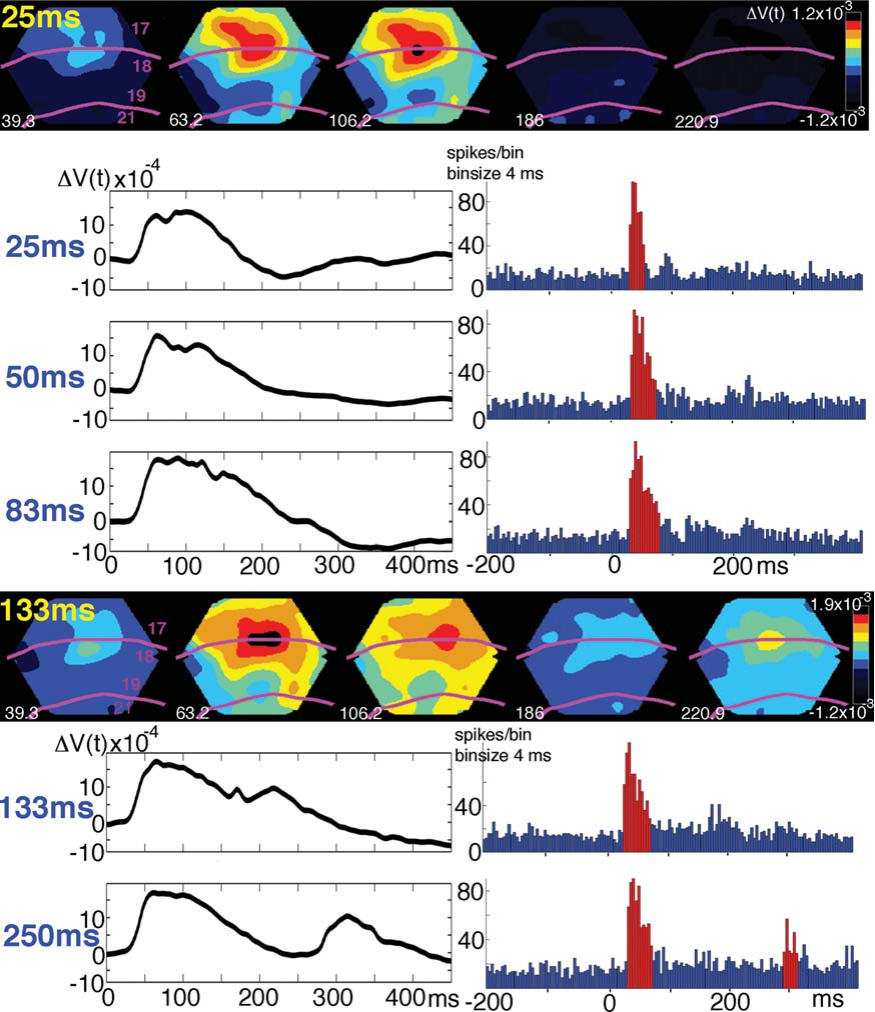
Voltage sensitive dye signal and multiunit activity for different stimulus conditions. Animal 5. The colored panels show the spatial distribution of the ΔV(t) for stimulus durations 25 ms and 133 ms at different times after stimulus onset as indicated in ms for each frame shown. The borders of the cytoarchitectural areas are marked with magenta; these borders mark also the vertical meridians. The black curves show the mean ΔV(t) from the square representation site at the cytoarchitectural border between areas 17 and 18 for each of the stimulus conditions. Note that all significant (p<0.01) ΔV(t) increases outlasts the stimulus by more than 100 ms. The post stimulus histogram from a single recording site at the square representation at the 17/18 border is shown corresponding to the stimulus duration indicated to the left. Note the truncation of the ON response at 25 ms giving a significant firing duration of 24 ms (significant firing p<0.01 marked with red bins), and the response to 50 ms giving a significant firing duration of 48 ms.

We presented a square defined by its luminance contrast in the center of field of view surrounded by a gray background. The duration of the square stimulus was 25 ms, 50 ms, 83 ms, 133 ms and 250 ms. We examined the relative changes in the membrane potentials of the neurons in layers I–III of the visual areas 17, 18 from the signals from the voltage sensitive dye. Compared to the condition of showing the gray screen, the square stimulus induced a relative increase of the population membrane potential ΔV(t). The ΔV(t) increase mapped for all durations, at the cytoarchitectural border separating area 17 from area 18 (**Fig. 2**). Figure 2 also shows that the statistically significant ΔV(t) changes at the square representation for all stimulus durations are delayed and much longer lasting than the stimulus. It is also apparent that the time interval between the ON peak of the ΔV(t) and the OFF peak of the ΔV(t) was longer than the stimulus durations 25 ms, 50 ms and 83 ms (**Fig. 2**). The spike trains recorded from the square representation were often short with small or no OFF responses for the short duration squares (**Fig. 2**).

### Multiunit ON and OFF responses

**Figure 3:**
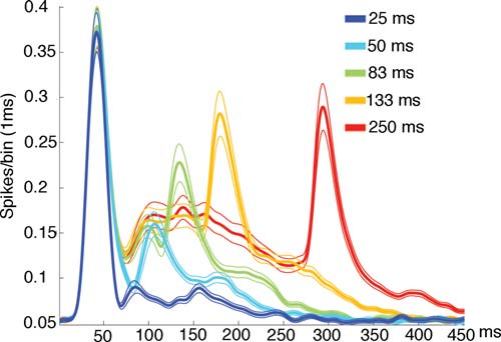
Mean firing rates. The mean firing rates across all animals. Note the significant truncation of 25 ms ON response and the significant dip in the firing rate just prior to the OFF response. The standard errors are shown as thin curves. The mean rate was calculated from all spike trains having statistically significant firing (p<0.01) to one or more of the stimulus conditions. Note that the dip just prior to the OFF response is reduced at longer stimulus durations and that the amplitude of the OFF response accordingly increases.

We recorded from 333 multunits in 86 penetrations of layers I–III of the cortex representing the square. Of these 99 fired significantly to one or more of the stimulus durations (**Fig. 3**). The mean amplitude of the ON response did not vary among the different durations. The duration of the ON response was significantly shorter for the 25 ms stimulus (**Fig. 3**). The timing of the ON peak did not change with the duration of the stimulus (**Fig. 3**). The amplitude of the OFF response, in contrast, increased non-linearly with the stimulus duration up to 133 ms (**Fig. 3**). The OFF responses were delayed. Compared to the timing of the peak of the ON responses, the latencies of the peak of the OFF responses were 22, 23, 17, 13, and 9 ms longer to the 25, 50, 83, 133, and 250 ms durations respectively. These prolonged latencies in the timing of the peak of the OFF firing were statistically significant for durations up to 83 ms (p<0.01). Thus the shorter the stimulus, the longer the OFF peak was postponed. Consequently, the peak to peak time interval between the ON and the OFF peak of the firing rate was significantly longer than the duration of the stimulus for stimulus durations up to 133 ms (p<0.01 two-tailed; t-test paired comparisons (n = 5) (**Table 1**).

**Table 1:** Peak latencies and peak to peak time intervals for multiunit firing rates and population membrane potentials (mean±stand. deviation).

	Stimulus duration				
	25	50	83	133	250 ms
Rate ON	42.0±1.1	40.4±1.0	41.8±1.1	41.8±1.4	41.8±1.3
Rate OFF	89.5±2.4	105.6±1.9	132.0±1.3	177.8±1.5	292.2±1.3
Rate duration	47.5±1.9	65.2±1.3	90.2±0.4	136.0±1.2	250.4±1.3
ΔV(t) ON	59.4±3.1	67.8±9.8	72.4±11.8	71.8±6.5	72.2±9.0
ΔV(t) OFF	120.6±10.9	139.6±2.8	166.5±16.7	218.6±10.9	330.45±23.4
ΔV(t) duration	61.1±12.9	70.7±9.4	99.6±21.7	146.2±19.3	258.2±23.2

The means and standard deviations were calculated from the averaged V(t) and averaged rates in each animal (n = 5).

### ON and OFF responses in the population membrane potentials

**Figure 4:**
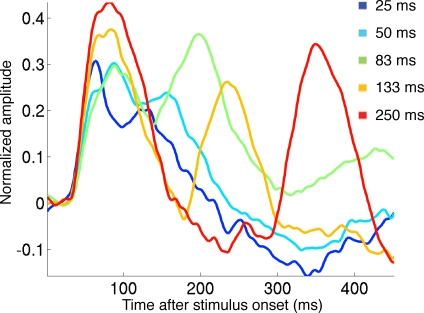
Mean relative population membrane potential. The ΔV(t) mean over all animals. The means were calculated from the relative means from each animal (1/5 Σ mean ΔV(t)rel, animal). The amplitude of the ON responses and OFF responses were significantly smaller and shorter for the 25 ms and 50 ms durations (p<0.01), compared to the other durations. The other differences in amplitudes were not significant. Note the reduction of the ΔV(t) amplitude at durations 133 ms and shorter, the truncation of the 25 ms and 50 ms ON responses, the delayed OFF responses and the negativity of ΔV(t) for the 250 ms duration in between the ON and OFF responses. The standard errors are shown in Fig. 6.

**Figure 5:**
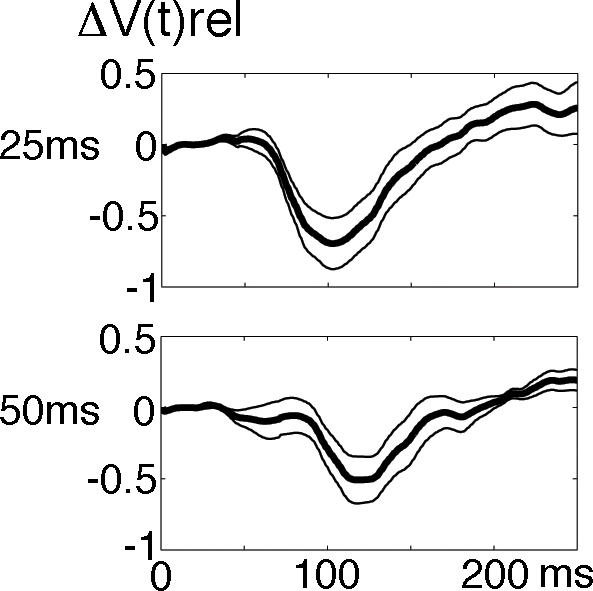
Non-linearity of the 25 ms and 50 ms ΔV(t). The normalized ON and OFF responses from the 250 ms condition were subtracted from the actually measured ΔV(t)rel for the 17/18 representation of the square. The curves were calculated as follows. First the ΔV(t)rel was calculated for each animal and stimulus condition (methods). Then the OFF response from the 250 ms condition was cut off the file at the time of stimulus offset. Then the ΔV(t)rel of the cut OFF response was adjusted so that its first 20 ms had a mean of 0. Then the such adjusted cut off OFF response was added to the ON ΔV(t)rel response after 25 ms, 50 ms, 83 ms and 133 ms. This gave four new files per animal giving the predicted values if the ON and OFF responses added linearly for each condition. Then the prediction file was subtracted from the actually measured ΔV(t)rel for each of the conditions 25, 50, 83 and 133 ms. The files were the averaged across animals for each condition and the standard deviation per time point calculated. The 25 ms and the 50 ms conditions then showed statistically significant deviation from linearity at 100 ms and 115 ms respectively (p<0.005).

**Figure 6:**
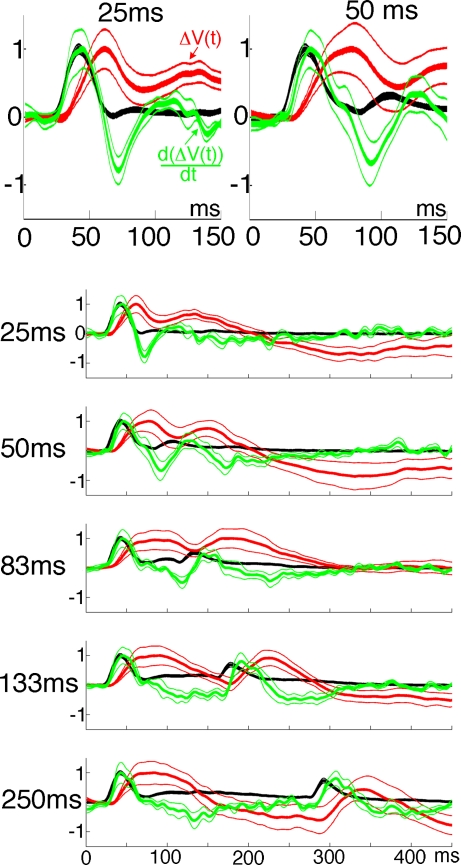
Mean relative firing rate, population membrane potential and d(ΔV(t))/dt. The mean relative instantaneous firing rate (black, calculated as ΔV(t)rel ), the mean ΔV(t)rel (red) and the mean time derivative of ΔV(t)rel d(ΔV(t)rel)/dt (green) calculated across all animals for each stimulus duration. The d(ΔV(t)rel) /dt is the relative change in the inward current in layers I–III (methods). The two top figures show the initial time-course of the relative firing rates, the ΔV(t)rel and the d(ΔV(t)rel)/dt at higher magnification.

The time course of the population membrane potentials in layers I–III at the square representation is shown in **Fig. 4**. The peaks of the ΔV(t) increases were much delayed compared to the ON and OFF peaks of the firing of action potentials (**Table 1**) (for explanation see discussion). We did a simple test for the non-linearity in the ΔV(t), demonstrating that the non-linearity was statistically significant and most pronounced for the 25 ms and 50 ms stimulus durations at 100 ms and 115 ms respectively (**Fig. 5**). The amplitudes of the ΔV(t) ON responses for the durations 25 and 50 ms were significantly reduced, compared to the ON responses of the stimulus durations 83, 133 and 250 ms (p<0.01 two-tailed; t-test paired comparisons, n = 5). Also the duration of the ON responses for the 25 ms and 50 ms durations were shorter than those for the 83, 133 and 250 ms (**Fig. 4**). The timing of the ON peak became progressively longer for stimuli of duration up to 83 ms (**Fig. 4** **and** **Table 1**). The amplitude of the ΔV(t) OFF peak was significantly smaller for the 25 ms and 50 ms stimuli compared to the 133 ms and 250 ms OFF peaks (**Fig. 4**,**6**). Also the OFF peak was postponed more compared to the stimulus offset for the short stimulus durations than for the longer stimulus durations. The OFF peak of the ΔV(t) had a latency longer than the ON peak by 36 ms, 22 ms, 11 ms, 14 ms and 8 ms for the durations 25, 50, 83, 133 and 256 ms respectively. These differences were significant for all stimulus durations (p<0.005 for each condition, t-test two-tailed, paired comparison). This also implied that the time interval between the ΔV(t) ON peak and the OFF peak was significantly longer than the actual stimulus duration for the 25 ms and 50 ms durations (p<0.005 and p<0.01 respectively, two tailed t-test) (**Table 1**).

### The time derivative of the population membrane potentials

Up to 80 ms after the start of the stimulus, the time derivative of the relative population membrane potentials, i.e. the d(ΔV(t))/dt was significantly correlated with the firing rate at the square representation (cross-correlation). This held for all stimulus durations (p<0.0001 for all durations) (**Fig. 6**). The lag that gave the highest r^2^ was 2±2 ms (mean±standard error for all stimulus durations). The d(ΔV(t))/dt, in the upper layers thus followed the changes in the ON firing rate with lag of 2 ms, but only up to 80 ms after the stimulus onset (**Fig. 6**). Or put in another way, the ON firing in layers II–III seemed to drive the changes in the population membrane potentials in layers I–III. From **Figure 6** one may further see that after the offset of the stimulus there is a strong and significant decrease in the d(ΔV(t))/dt. The decrease of the d(ΔV(t))/dt is simultaneously with a dip in the firing rate. The (negative) amplitude of the dip in the firing rate and the dip in the d(ΔV(t))/dt are most marked for the 25 ms duration and decrease gradually with increasing stimulus durations.

The delay of the d(ΔV(t))/dt increase with respect to the OFF firing is, due to the preceding negativity of the d(ΔV(t))/dt, much longer than the 2 ms lag of the d(ΔV(t))/dt to the ON firing. However, the d(ΔV(t))/dt OFF increase was also correlated significantly to the OFF firing in all conditions (p<0.001). The estimated lag was 20 ms for the stimulus duration of 25 ms and decreased to 19 ms, 18 ms, 15 ms, and 10 ms for the stimulus durations 50 ms, 83 ms, 133 ms, and 250 ms respectively (see also **Fig. 6**). The delay of the peak of the ΔV(t) OFF response with respect to the offset of the stimulus decreased from 96 ms for 25 ms duration to 80 ms for 250 ms duration. This decrease then corresponds roughly to the decrease in the lag of the d(ΔV(t))/dt with respect to the OFF firing.

### Encoding of stimulus duration

**Figure 7:**
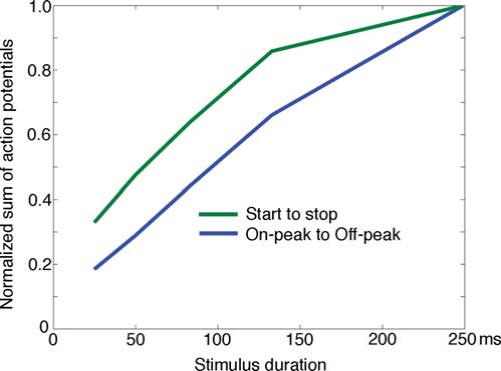
Predicting the stimulus duration from the spike trains. For all spike trains having significant firing (p<0.01) the number of spikes were summed from the first significant bin to the last significant bin (irrespective if the firing between the ON and OFF responses was significant) for each condition. The maximal sum for each animal was set to 1.0 and the other sums normalized accordingly. This gave the green curve: start-stop. The blue curve was calculated similarly, but such that the sum of spikes was only taken between the peak of the ON response and the OFF response: peak-to-peak.

The ON peak to OFF peak time interval would encode the stimulus duration as being considerably longer than the actual duration for stimuli less than 100 ms (**Fig. 4**, **6**; **Table 1**). There are several remaining possibilities of encoding the stimulus duration, but we only examined two. The first was that the stimulus duration could be encoded by the number of spikes from the start of the significant firing to the last significant spike bin (1 ms) [36]. The second was that the stimulus duration could be encoded by the number of spikes between the ON and OFF peaks of the firing. As seen from **Figure 7** the summation of the number of spikes between the ON and the OFF peaks gave the best estimate.

## Discussion

The ON firing as well as the OFF firing preceded the relative increases in the population membrane potentials in layers I–III at the cortical location of the square stimulus. The time derivative of the population membrane potentials, the d(ΔV(t))/dt, was strongly correlated with the changes in the firing rates for the ON response with a lag of a few ms. The d(ΔV(t))/dt was also correlated with the firing of the OFF response, but with a longer delay. This delay decreased as the duration of the stimulus increased. The duration of the ON response was shorter for short stimulus durations of 25 ms and 50 ms (ΔV(t)) than for longer durations. The OFF response in firing as well as in the ΔV(t) was preceded by a short decrease in the firing rate and a strong decrease of the d(ΔV(t) )/dt. The multiunit OFF responses were postponed by up to 25 ms compared to the ON responses and the ΔV(t) OFF signal was further postponed, mostly for the short durations. The OFF responses increased non-linearly in amplitude as the stimulus duration increased. These non-linearities in the appearance of the OFF response made the time interval between the ON and OFF responses a poor estimate of the stimulus duration.

### Relation between firing and changes in population membrane potentials

That the ON firing precedes the relative increase in the population membrane potentials was reported recently [37], but the time relations between the firing rate and the relative population membrane potentials have not been reported. The ΔV(t), the difference signal between the gray screen condition and the presentation of the luminance square, is the relative change in the membrane potentials of the neurons in layers I–III as described in the introduction and methods sections. The time derivative of the ΔV(t), the d(ΔV(t))/dt, may, if one regards all stained membranes of cells in layers I–III as one single compartment, be an indicator of the inward currents of the populations of cells in layers I–III as shown in the introduction. An overall increase in excitation would, especially if the population of cells are only moderately depolarized, increase the d(ΔV(t))/dt and subsequently increase the ΔV(t). For example, at the cortical representation of the square, the increase of firing rates to the onset of the square stimulus was followed by a similar increase of the d(ΔV(t))/dt with a lag of 2 ms (**Fig. 6**). That the (net) increase in d(ΔV(t))/dt followed the (net) increase in firing rate, and not the other way round, has a simple explanation. As we were recording the ΔV(t) from large populations of neurons, and as every neuron firing action potentials sends synapses to at least 2000–3000 other neurons [38]–[40], the number of neurons depolarized by the firing neurons is much larger than the number of neurons firing. This is not to deny, that logically, the neurons firing must have been shortly depolarized prior to their firing, but that this depolarization from a population perspective was so small that the depolarization of neurons in their surround totally dominated the ΔV(t) [31]. Thus, the bulk of the inward currents of axons and dendrites in layers I–III and hence the d(ΔV(t))/dt signal will shortly lag the firing and the ΔV(t) will lag the d(ΔV(t))/dt in the layers I–III. In our data, the ON firing and OFF firing in layers II–III seem to drive the ΔV(t) ON and OFF responses. This means that the neurons firing at the cortical representation of the square depolarizes many other neurons here which is detectable as increases in d(ΔV(t))/dt and ΔV(t).

One may think that the truncation of the ON response observed for the short duration stimuli of 25 ms and 50 ms (**Fig. 3** **and** **4**) was due to refractoriness of the neurons or to depressing synapses. However, the maximum ON firing rate was identical for all stimulus durations and thus not especially high for the short duration stimuli. It is therefore unlikely that refractory periods and depressing synapses can explain the truncations and the delayed onset of OFF firing.

### Decreases in the inward currents

In order to explain our results, we propose that the offset of a stimulus elicits a decrease of the inward currents. We observed a dip in the firing rate and a stronger dip in the d(ΔV(t))/dt after the stimulus offset, but prior to the OFF response. Both dips were time-locked to the stimulus offset with a short latency (28–33 ms) and preceded the OFF response for all stimulus durations (**Fig. 6**). So, the offset of the stimulus was associated with a negative d(ΔV(t))/dt. This negativity we interpret as a decrease in the net inward current to the cell compartment (equation 3, introduction). The inward current in equation 3 is defined as an influx of positive ions into the cell compartment (building up positive charge inside the cells). A decrease in the net inward current means a decrease in the influx of positive ions (Na^+^ and/or Ca^2+^) into the cell compartment, or an increase in the efflux of positive ions (K^+^) from the cell compartment. A third possibility is influx of negative ions (Cl^−^), which will decrease the positive charge. The following observations suggest that the decrease in the net inward current could be due to the influx of negative ions (Cl^−^) or the efflux of positive ions (K^+^), i.e. inhibition.

Depending on the population membrane potentials in layers I–III and hence the inhibitory drive at the stimulus offset, inhibition will act non-linearly on the firing rate OFF responses and on the ΔV(t) OFF response. The inhibitory drive is the difference between the average population membrane potential at each cortical point and the reversal potentials for the inhibitory currents. Under strong net excitation, as during the firing of the ON response, the d(ΔV(t))/dt dip should be strong, and indeed it was. Under weak excitation of the population membrane potentials, the d(ΔV(t))/dt dip should be weak, and indeed it was. As the d(ΔV(t))/dt dip was time- locked to the stimulus offset and preceded the OFF response, this indicates that inhibition preceded the excitatory OFF response. At short stimulus durations the stimulus offset occurred during the firing ON response at a time when the excitation was near maximum, and hence the inhibitory drive also near maximum. This could explain the truncation of the multiunit ON response and the membrane potential ON response for short stimulus durations (**Fig. 3**, **4** **and** **6**).

The inhibitory effect should diminish with increasing duration of the stimulus in accordance with the prevailing population membrane potential at the time of stimulus offset. At short stimulus durations the strong inhibitory drive preceding the (excitatory) OFF response should delay the OFF response in firing as well as in ΔV(t). This effect should diminish with the decrease in excitation of the population membrane potential, which was also observed (**Fig. 4** **and** **6**). Moreover, as the negative amplitude of the d(ΔV(t))/dt dip decreased with the population membrane potential at the time of stimulus offset for longer stimulus durations, the non-linear effects of the decreasing inhibition should increase the amplitude of the OFF responses. This then would explain why the peak amplitude of the OFF firing and OFF ΔV(t) is the smallest for the shortest stimulus duration and why the amplitude increases non-linearly with increasing duration [13].

Using similar stimuli as our stimuli and stimulus durations of 29–39 ms, Hirsch et al. [14] measured the membrane potentials and firing of single neurons in the cat area 17. Layer IV simple and complex cells changed their membrane potential and firing quite similarly to the neurons in the lateral geniculate nucleus, i.e. outlasting the stimulus duration [10], [41]. Neurons in layers II–III had much shorter firing that in a few cases was followed by an inhibition truncating the ON response. Our results from the upper layers at the population level are in harmony with these findings. The results of Hirsch et al. [14] further suggest that the inhibition also in our case may be intra-cortical and specific to layers II–III. Furthermore, these intracellular data also indicate that the truncation of the ON response was not due to reduced excitation and spike adaptation. Whenever a new stimulus is introduced, there is probably a strong increase in both excitatory and inhibitory currents simultaneously during the ON response [42]–[46]. A sudden early offset of the stimulus, on the top of the ON response may then elicit a strong increase in inhibitory currents, because the inhibitory drive is near maximal. Whether this offset inhibition is because the OFF afferents from layer IV target the network in a different way than the ON afferents do, or it is due to the network properties in layers II–III or both is not known.

### Stimulus duration and visual persistence

If the brain uses the peak to peak time interval between the ΔV(t) ON and the ΔV(t) OFF, this will overestimate the duration more than if the brain relied on the firing of neurons in layers II–III (**Table 1**). Integration over longer time intervals, as for example from the first statistical increase of the ΔV(t) to the last time-point of significance would do even worse as the ΔV(t) outlasts the ΔV(t) OFF peak by some variable 80–180 ms (**Fig. 4** **and** **6**).

As we did not examine the firing and relative membrane potentials in layers IV–VI, we can only comment on the ON-OFF firing pattern of the neurons in layers II–III. The ON peak firing was delayed by 42 ms, but this delay was stable for the different durations. Unfortunately the timing of the OFF peak firing varied non-linearly with the stimulus duration, leading to overestimation of the stimulus durations up to 83–133 ms (Table 1). Another strategy would be to use the OFF firing amplitude as an indicator for stimulus duration [13]. However for the large population of multiunits examined by us, the OFF amplitude increased non-linearly with stimulus durations, but then stopped to increase for stimulus durations longer than 133 ms (**Fig. 3**). The best estimate of the stimulus durations was from the sum of action potentials integrated between the ON peak and the OFF peak of firing. This estimate did better than integration of action potentials from the statistical onset of firing to the last significant firing, probably because the statistically significant firing outlasts the OFF peak by up to 300 ms (**Fig. 3**). The almost linear relation between stimulus duration and the sum of action potentials between the ON and OFF peak (**Fig. 7**) could be due to the offset inhibition decreasing the firing of the ON and OFF responses as well as the firing in between and thus compensating for the relative longer integration interval for the shorter durations. The firing patterns in layers II–III may be of importance for judgments of visual persistence and stimulus durations as layer III is the output layer to other cortical areas [19], [20].

What animals and humans actually use as a strategy for computing the duration of a transient stimulus is still not clear. Humans at least do perceive a visual persistence more pronounced the shorter the stimulus lasts [47], and humans actually have difficulties discriminating the duration of stimuli when these last less than 100 ms [5], [6].

## Materials and Methods

All experimental procedures were approved by the Stockholm Regional Ethics Committee and were performed according to European Community guidelines for the care and use of animals in scientific experiments. Recordings were performed in 5 adult, female ferrets. The ferrets were initially anesthetized with Ketamin (15 mg kg^−1^) and Medetomidine (0.3 mg kg^−1^) supplemented with Atropine (0.3 mg kg^−1^). After the initial anaesthesia the ferrets received a tracheotomy and were ventilated with 1∶1 N_2_O∶O_2_ and 1% Isoflurane. The expiratory pCO_2_ was kept within the 3.5–4.3 KPa range and ECG and EEG was recorded continuously during the experiment. A craniotomy was made exposing left hemisphere visual areas 17, 18, 19, and 21, and adjacent temporal and parietal lobe areas. The craniotomy was covered with a chamber affixed to the skull with dental acrylic. Animals were paralyzed with pancuruonium bromide (0.6 mg kg^−1^), the left eye was occluded, and the right eye had its pupil dilated (1% atropine), the nictating membrane retracted (10% Phenylephrine) and the eye was then fitted with a zero power contact lens.

### Stimulation and voltage sensitive dye recordings

#### Setting the spatial relation between the display screen and the animal

A reverse ophthalmoscope was used to record the position of the optic disk and centre a video monitor to the area centralis. Known cortical landmarks were then used to guide a single electrode penetration at the estimated crossing of the vertical and horizontal meridian. The action potentials of single/multiple neurons were recorded with a thin tungsten electrode (impedance range: 800 kOhms to 1.1 MOhms; FHC ®, Boudain, ME). This electrode was used to find the crossing of the vertical and horizontal meridian, i.e. the center of field of view. Then the receptive field of the unit was mapped with the m-sequence [48]. If the receptive field was 1.5° or less in diameter, and the electrode penetration was at the crossing of the vertical and horizontal meridian (assessed with vertical and horizontal line stimuli), the screen's position was adjusted to present the center of the stimulus within the receptive field. This electrode position on the cortex was then called the *center of stimulus representation*.

#### Dye Staining and recording

After the initial electrophysiology, the exposed cortical surface, containing visual areas 17, 18, 19 and 21 was stained for 2 hours with the voltage sensitive dye RH1838 (0.53 mg ml^−1^) (Optical Imaging, Rehovot, Israel) (**Fig. 1**). This dye stained all layers of the cerebral cortex. Due to attenuation of the photons, the signal reaching the detectors will stem mainly from the upper, layers I–III [29], [33]. Each detector channel monitored a small circular cortical area of 150 µm in diameter.

After staining, the cortex was rinsed with artificial cerebrospinal fluid, the chamber was filled with silicon oil and sealed with a cover glass. Imaging was centred on the initial recording site and acquired using a 464-channel photodiode array, (H469-IV WuTech Instruments Gaithersburg, MD) through a macroscope fitted with a 5× objective (Red Shirt New Haven, CT). Images were acquired at a rate of 0.616 ms per frame. The stimulus presentation was synchronized to the ECG signal, and respiration stopped during stimulus presentation (2 s).

#### Stimulation

The stimuli were presented every 15 s in a pseudo-random order on a video monitor with a refresh rate of 120 Hz located 57 cm in front of the animal. The stimulation was controlled by a VSG series IV system (Cambridge Research Systems, Kent UK). The control condition was a uniform gray screen with a luminance of 30 cd.m^−2^. The gray screen was on between trials. The stimulus condition was a white square, 2.5° by 2.5°, of 120 cd.m^−2^, presented in the center of field of view on the gray background of 30 cd.m^−2^. The durations of the square stimulus were 25 ms, 50 ms, 83 ms, 133 ms and 250 ms. Each of the six conditions was repeated 50 times for the voltage sensitive dye recordings and the multiunit recordings.

### Electrophysiology

After the dye measurements the action potentials of multiunits were recorded with a four shank multi-electrode with a spacing of 200 µm between shanks. Each shank had four recording sites spaced of 200 µm apart (Michigan Probes, University of Michigan, Center for neural communication and technology). The impedance of the recording sites ranged from 0.296 MOhm to 0.367 MOhm. The shanks were positioned such that the upper recording sites were just at the cortical surface. This implied that all recordings were from layers I–III [49]. First the multielectrode was placed with one of the shanks at the center of object representation. The RF for each of the leads were then mapped again (m-sequence) [48], [50]. Only if the initial responses could be reproduced the electrophysiology was judged valid and the animal included in the experimental series. We calculated the temporal weighting response function [51] in 15 animals during the receptive field mapping before and after the dye staining. There were no statistically significant differences at any time point of the temporal weighting function (p>0.2; n = 15). After the second, post dye, receptive field mapping, the electrode was moved to different positions within the exposed part of area 17 and area 18. Throughout the experiment the EEG was recorded from the scalp. The EEG was dominated by regular delta and theta waves.

### Cytoarchitectonics and reconstruction

At the end of the experiment, three vertical needle marks were made around the recorded area, the animals were sacrificed (pentobarbital) and perfused transcardially with 4% paraformaldehyde. First three additional horizontal needle marks were made in the block dissected from the brain. The block was sectioned and alternate 50 µm sections were stained for Nissl and cytochrome oxidase. Areal borders were then reconstructed using the cytoarchitectonic borders marked on each section [49]. Then the images of the sections were reconstructed to a three dimensional volume of the posterior part of the brain using an in house computer program. The cytoarchitectural borders were mapped onto the image of the cortical surface. The reconstruction was fitted to the pictures of the operative field and voltage sensitive dye recording sites, to match the electrode penetrations. The reconstruction provided a mapping of the cytoarchitectural borders between the four visual areas 17, 18, 19, and 21. As the cytoarchitectural border between areas 17 and 18 marks the position of the vertical meridian, we could by this independent information evaluate whether the initial electrode penetrations, for the localization of the crossing between the vertical and horizontal meridian (**Fig. 1**), indeed were localized along the vertical meridian. Similarly, we evaluated whether the representation sites of the square stimulus were overlapping the cytoarchitectural border between areas 17 and 18.

### Data Analysis

All voltage sensitive dye signals were analyzed in terms of fractional fluorescence [37]. In brief, the signal from the background condition was subtracted from the signal of the stimulus condition and divided by the fluorescence with the screen off to yield the fractional fluorescence, referred to here as ΔV(t). This subtraction was done in order to remove pulse artefacts. Although the stimulus presentation was synchronized with the ECG signal, this does only guarantee that the first ECG spike is aligned for the stimulus and background trial, as the later ECG spikes may diverge more and more. To remedy this we used the procedure described in [33], [34]. This procedure efficiently removed the pulse artefact as judged by an analysis of the files, the autocorrelation function and the Fourier power spectrum as also documented in [34].

ΔV(t)_xy_ is the difference in fluorescence to the stimulus minus the fluorescence to the baseline gray screen, divided by the fluorescence obtained in darkness F_0 xy_ (i.e. the fluorescence when no light is entering the eye of the animal, black display and usual darkness in the surrounds). The F_0 xy_ was determined four times, at the start, at the end and two times in between the sessions. For one detector channel x, y:
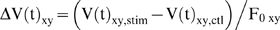



In which stim is a stimulus condition, ctl is the condition with only the gray screen presented. Usually 16 to 30 ΔV(t)_xy_ files were averaged by adding the files and dividing by the number of files. In the text this average time function of the relative membrane potentials is referred to as simply ΔV(t). Using the amplitude fluctuations in the pre-stimulus interval to define the noise level for each photodiode channel, the ΔV(t)_xy_ was thresholded at p<0.01 of being noise (p<0.01 one-sided, as only ΔV(t) increases occurred in response to the stimulus conditions). In this we assumed the amplitude fluctuations to be not significantly different from a Gaussian distribution. A threshold of estimated p<0.01 was set for each photodiode detector channel and divided by the number of channels (464) to give the Bonferroni corrected value of p<0.01 which was used for determining the statistical significance.

The relative amplitude was calculated for the post-stimulus interval, i.e., from 0 ms to 400 ms after the start of the stimulus as the ΔV(t)_xy_ divided by the overall maximal amplitude, i.e.
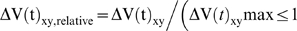
In the text, the index xy is suppressed and the time course of ΔV(t)_xy,relative_ is referred to simply as ΔV(t)rel.

In order to calculate significant responses in the spike trains, a Poisson distribution was fitted to the spike trains in the pre-stimulus period *and* spikes from the background trials. Spike trains passing both the criterion of having significantly increased discharge rate compared to the pre-stimulus period of p<0.01 *and* increased rate compared to the background condition of p<0.01, were considered statistically significant periods of firing.

### The voltage sensitive dye signal and its time derivative, definitions

In vitro the dye signal, V(t), is a linear function of the membrane potential [27]–[29]. However, as the absolute dye signal depends on the amount of staining, one divides the raw signal by F_0, xy_. Further, the dye signal must be calibrated by intracellular recordings/patch clamping. It is not possible in vivo to calibrate the dye signal in large populations of neurons. Furthermore, in vivo, the photons from deeper layers of cortex are attenuated and those from the upper layers are scattered. In addition, the in vivo signals, V(t)_xy,stim_ and V(t)_xy,ctl_ have a pulse artifact. The pulse artifact can in practice be removed (see above). Still given this, the signal may also be subjected to equipment noise and fluctuations in the number of photons due to variations in the illumination source.

Still if one assumes the noise sources are invariant and the pulsation artifact removed, it is not possible to measure depolarization and hyperpolarization in vivo. In the strict sense depolarization is an increase in the *absolute* value of the membrane potential of a cell. This means that depolarization is defined from the resting potential, that is the membrane potential of a neuron without any synaptic input. Consequently the definition of depolarization and hyperpolarization will not work in vivo. The ΔV(t)_xy_ is a difference signal between the signal introduced by the background and the square+background that is made relative due to the division by the resting light intensity, F_0_. If the ΔV(t) is >0 it means that the cortex from which the signal originates is relatively more depolarized during the stimulus condition, than during the condition when only the background is exposed to the animal. If the ΔV(t)<0 the cortex during background condition is relatively more depolarized than it is during the stimulus condition. The ΔV(t)_xy_ will consequently indicate changes in the deporlarization direction and changes in the hyperpolarizaing direction, provided that the pulse artifact is removed and the noise is identical in the two conditions. Furthermore as the component from glia cells is moderate and has a much slower time course compared to the neuronal changes in membrane potentials [52]–[57], fast changes of ΔV(t) may be ascribed to the neurons. That the ΔV(t) is a reliable measurement of the relative changes in population membrane potentials of layer I-III neurons is also verified by simultaneous in vivo measurements of the V(t) and the membrane potentials of neurons in layers II and III [29]–[33]. Therefore, we use the term relative changes in population membrane potential for ΔV(t) increases under the above conditions.

If one regards the cell with stained membranes in layers I–III as one single compartment, then the time derivative of the ΔV(t), the d(ΔV(t))/dt is related to the rate with which current flows into the cells of layers I–III. If Vm is the membrane potentials of a set of neurons, then dVm/dt Cm = dQ/dt. Cm is the average total capacitance of the neurons and Q is the average charge. The dVm/dt is dependent on the membrane specific conductances for inhibition, excitation and leak currents, and the differences between the membrane potentials and the reversal potentials. So one may regard the d(ΔV(t))/dt as a indicator of the weighted relative changes in the net inward currents in layers I–III. We calculated the d(ΔV(t))/dt in each interval t−0.616 ms to t+0.616 ms. If the conductance to chloride^−^ increases under inhibition, this will decrease the d(ΔV(t))/dt [42], [57]. The driving force of this inhibition will be the strongest if the cells of the population are already strongly depolarized. This is due to the fact that the driving force is the difference between the actual (average) membrane potential and the reversal potentials for Cl^−^ and K^+^. As the reversal potentials for these ions are more negative than is the resting potential, the driving force of inhibition will be the largest when the neurons are strongly depolarized.

The square representation at the area 17/18 cytoarchitectural border was defined as the cortical space within a radius of 600 µm of the center of representation. We defined the ON and OFF responses in the spike trains as the statistically significant firing giving rise to the highest and next to highest peaks in the mean of the instantaneous firing rates at the cortical representation of the square in one animal.
